# Enhanced electronic-transport modulation in single-crystalline VO_2_ nanowire-based solid-state field-effect transistors

**DOI:** 10.1038/s41598-017-17468-x

**Published:** 2017-12-08

**Authors:** Tingting Wei, Teruo Kanki, Masashi Chikanari, Takafumi Uemura, Tsuyoshi Sekitani, Hidekazu Tanaka

**Affiliations:** 1 Institute of Scientific and Industrial Research, Osaka Universit, Ibaraki, Osaka, 567-0047 Japan; 2 Faculty of Science, Kunming University of Science and Technology, Kunming, 650093 China

## Abstract

Field-effect transistors using correlated electron materials with an electronic phase transition pave a new avenue to realize steep slope switching, to overcome device size limitations and to investigate fundamental science. Here, we present a new finding in gate-bias-induced electronic transport switching in a correlated electron material, i.e., a VO_2_ nanowire channel through a hybrid gate, which showed an enhancement in the resistive modulation efficiency accompanied by expansion of metallic nano-domains in an insulating matrix by applying gate biases near the metal-insulator transition temperature. Our results offer an understanding of the innate ability of coexistence state of metallic and insulating domains in correlated materials through carrier tuning and serve as a valuable reference for further research into the development of correlated materials and their devices.

## Introduction

As a representative transition metal oxide with correlated electrons, vanadium dioxide (VO_2_), has attracted considerable research attention because of its versatile properties. Primarily, this material shows a dramatic orders-of-magnitude resistivity change associated with the metal-insulator transition (MIT) at room temperature^1,2^ and a distinct contrast in the optical properties^3^ of the insulating and metallic phases. A variety of applications have been suggested based on these characteristics, such as multistate memory utilizing local domain transitions^4–6^, oscillators^7–10^ and electrical/optical switching devices^11–14^. Among these applications, field-effect transistors (FETs) to control of the MIT by a gate bias, so-called Mott transistors (Mott-FETs), have been particularly attractive over the past decade^15–18^. The field-triggered transport modulation ratio, however, has demonstrated comparatively low values of less than 0.6% for fast switching (or ~5% for slow switching)^19–22^. Much effort has been devoted to enhancing the resistance modulation, mainly focusing on inducing a large carrier density *via* a high electric field using a high-dielectric-constant (high-*k*) gate dielectric. The recent development of Mott-FETs has been attempted by fabricating an electric double-layer transistor (EDLT) with a strong electric field by applying to VO_2_ channels^23,24^, while the existence of issues in the chemical reaction remain under debate^25^. In our previous report using VO_2_ thin-film-based FETs, a high-*k* inorganic Ta_2_O_5_/organic polymer parylene hybrid solid gate insulator^22,26,27^ was demonstrated, leading to reversible and prompt electrical-transport modulation owing to reduced interface deterioration and a high dielectric constant in the bi-layered gate insulator. As another new approach, the use of nanostructured channels is promising because the MIT sensitivity is highly responsive to sizes comparable in scale to the electronic phase domains, resulting in a dramatic resistance jump due to electronic avalanche effects^28–30^.

In this study, we report an enhanced on/off ratio by using epitaxial VO_2_ nanowire-based FETs with a high-*k* Ta_2_O_5_/parylene hybrid solid gate insulator. In contrast to thin-film channels^22^, nanowire channels have superior sensitivity for transport modulation, resulting in an approximately ten-fold higher resistance modulation than that in thin-film channels. The enhancement in the resistance modulation is derived from expansion of metallic nano-frictions in an insulating matrix due to carrier accumulation driven by applying an electric field.

## Results

### Formation of VO_2_ nanowire-based FET devices

**Figure 1:**
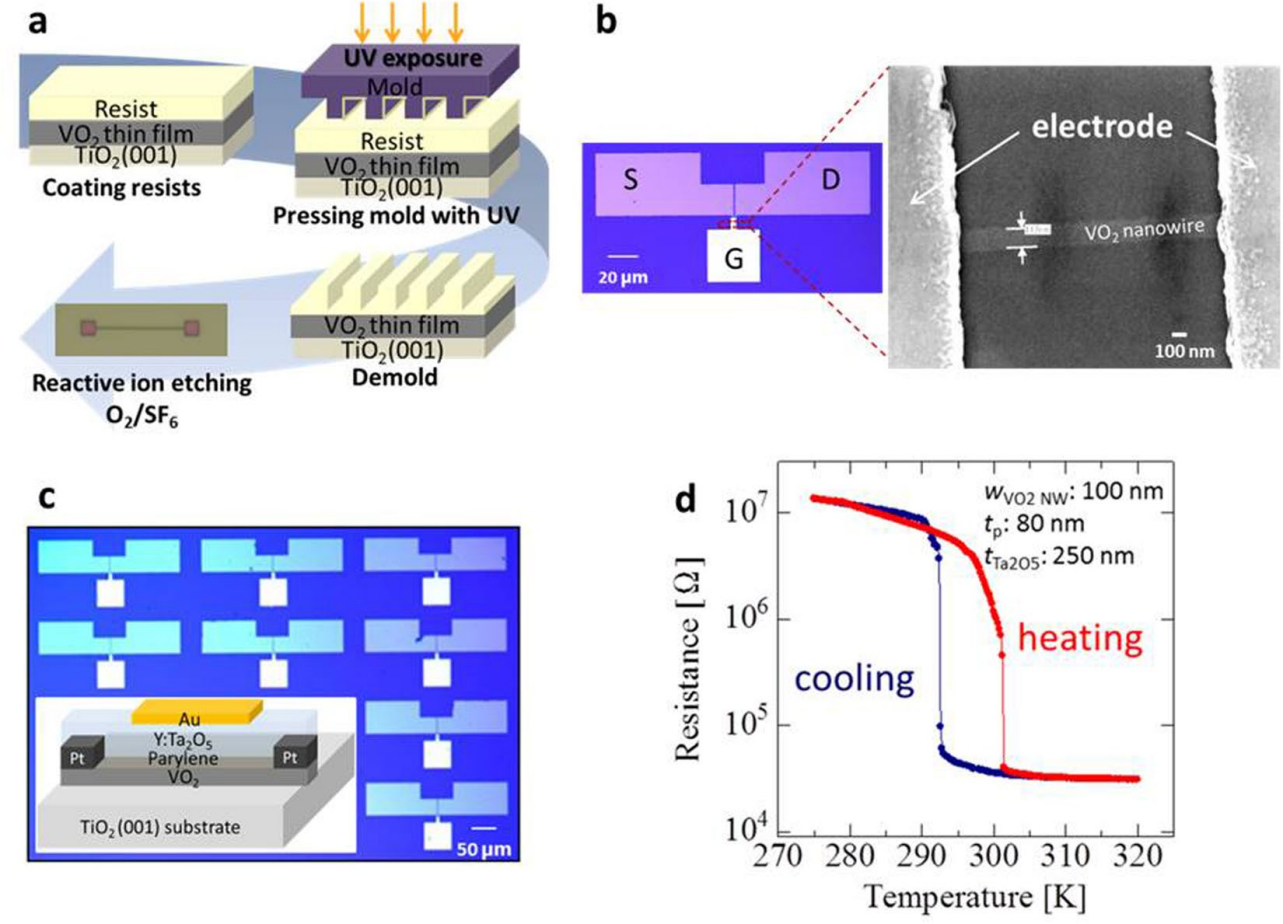
Device structure and characterization. (**a**) VO_2_ nanowire fabrication process using UV-NIL. (**b**) Representative VO_2_ nanowire-based field-effect transistor structure and morphology of the VO_2_ nanowires, as determined by scanning electron microscope (SEM). (**c**) Optical microscopy image of the VO_2_ nanowire-based FETs with a Y-doped Ta_2_O_5_/parylene hybrid gate dielectric; the inset shows a schematic illustration of the device structure. (**d**) Dependence of the resistance change on the temperature of an as-fabricated VO_2_ nanowire-based FET.

This work begins with synthesis of epitaxial single-crystal VO_2_ nanowires using the ultraviolet-nanoimprint lithography (UV-NIL) technique. The fabrication process is depicted in Fig. 1a. First, a resist is spin-coated onto epitaxial VO_2_ thin films, and then, a mold pattern containing nanowires is pressed into the resist under UV light. After a demolding process, the nanowire pattern is transcribed onto the VO_2_ thin films. Subsequently, the nanowire patterns are formed by reactive ion etching (RIE). The image on the right in Fig. 1b shows a scanning electron microscope (SEM) image, which confirms the morphology with high precision. The electrode patterns shown in the image on the left in Fig. 1b were obtained by a conventional photolithography technique, and the size-controllable VO_2_ nanowire-based FETs with 100- to 300-nm-wide and 8-nm-thick nanowires, as identified by atomic force microscopy (AFM), were manufactured over a large area (~200 devices on a single chip), as shown in Fig. 1c. Temperature-dependent resistance measurements were performed to investigate the MIT behaviour. The results are shown in Fig. 1d. An abrupt MIT as a function of temperature was achieved in all of the as-synthesized nanowire-based FETs with different nanowire widths, showing a different behaviour from that of film-based devices, which undergo a gradual MIT (see Fig. S1, Supplementary Information). This dramatic change in the nanowire resistance indicates the high MIT sensitivity to small temperature variations. In the typical device configuration, the VO_2_ nanowire width is 100 nm, the parylene layer is 80 nm thick, and the overlay Y-doped Ta_2_O_5_ layer is 250 nm thick. For comparison, we also fabricated VO_2_ film-based FETs with similar hybrid gate insulator thicknesses. The fabrication of the film-based FETs was described in detail in our previous paper^22^.

### Resistance switching behaviour in VO_2_ nanowire-based FET device

**Figure 2:**
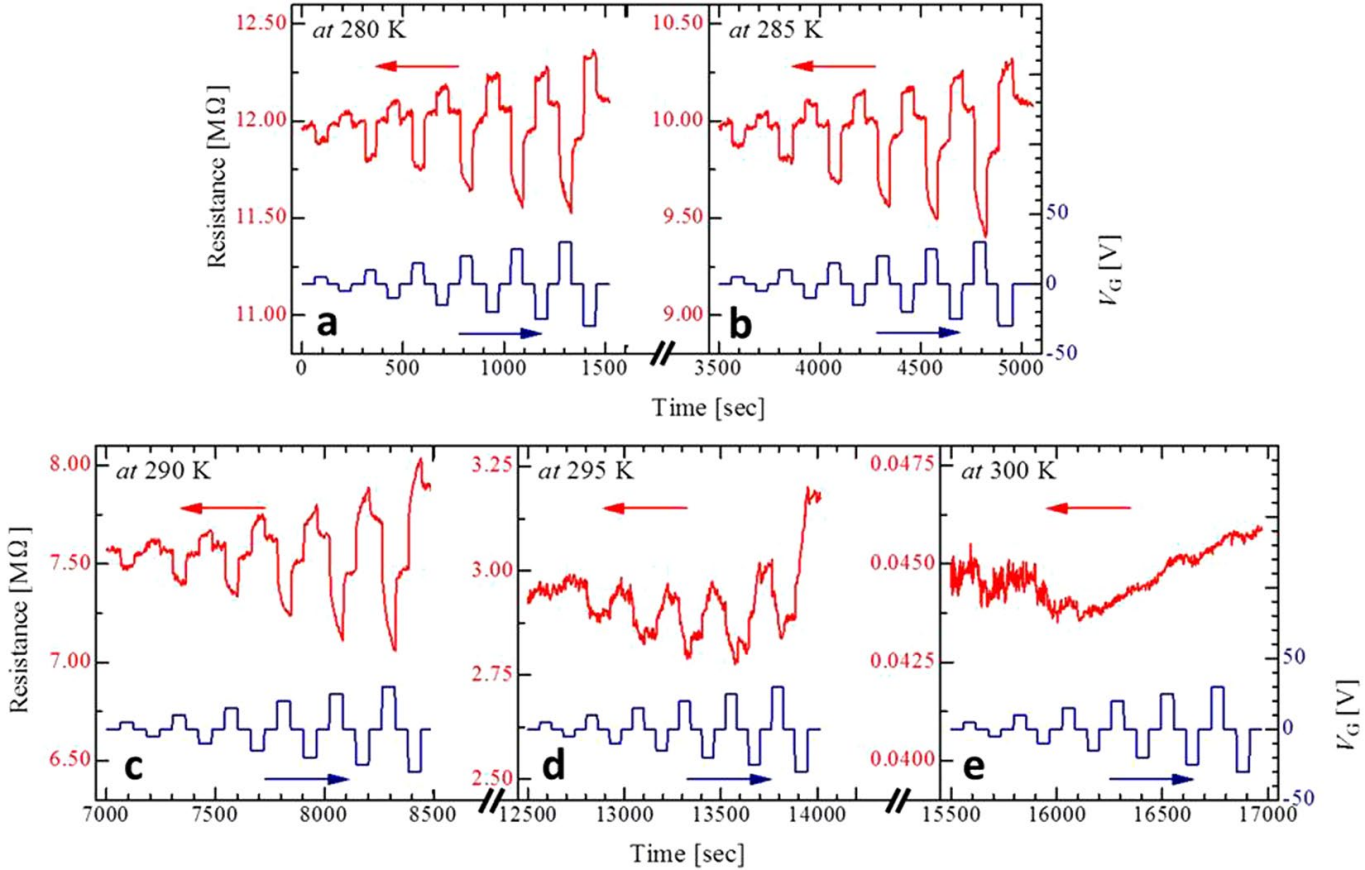
Resistance switching behaviour by applying a gate bias. (**a–e**) Resistance response versus gate bias in the VO_2_ nanowire channel near *T*_MI_ from 280 to 300 K.

**Figure 3:**
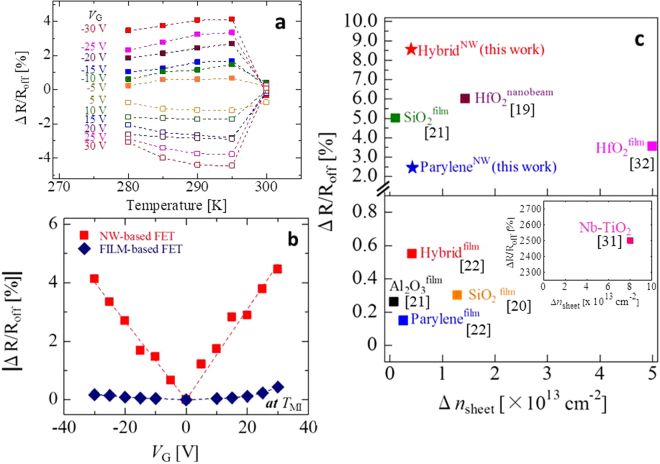
Resistive modulation ratio by an electric field in VO_2_ nanowire and thin-film channels. (**a**) Resistance modulation efficiency as function of temperature at various gate biases applied in VO_2_ nanowire channels. (**b**) Comparison of the absolute resistance modulation ratios for the nanowire-based and thin film-based FETs. (**c**) Comparison of studies of VO_2_-based FETs with various solid gate insulators from other papers, represented by the resistance modulation efficiency and induced sheet carrier density. The parylene-gated nanowire FET data are introduced in Fig. S5, Supplementary Information.

To investigate transport modulation in the VO_2_ nanowire channel, the resistance was measured under various applied gate biases (*V*_G_ = 0 to ±30 V at intervals of 5 V) as a function of time near the transition temperature (*T*_MI_), as shown in Fig. 2a–e. Each resistance response cycle followed the same trend under various gate biases, including two main features: a flexible modulation in the channel resistance in response to the magnitude of the gate bias. Second, the resistance showed an abrupt drop and rise under positive and negative gate biases, respectively, from 280 K to 295 K in the insulating region. This behaviour is consistent with that of thin-film-based FETs^22^ (see Fig. S2a–f in Supplementary Information). When the resistance modulation efficiency is defined as follows:

1 $$\frac{{\rm{\Delta }}R}{{R}_{off}}=\frac{{R}_{on}-{R}_{off}}{{{\rm{R}}}_{off}}\times 100 \% $$

where *R*_on_ and *R*_off_ are the resistances with and without a gate voltage, respectively, Δ*R*/*R*_off_ is considerably enhanced in the VO_2_ nanowire channels in comparison with that in the thin-film channels^22^. Figure 3a shows the summary of Δ*R*/*R*_off_ estimated from Fig. 2a–e. The maximum resistance modulation ratio is approximately 8.58% at *V*_G_ = ±30 V just below the phase-transition temperature of 300 K in the heating process, defined as the point of maximum d*R*/d*T*, whose Δ*R*/*R*_off_ is over 10-fold higher than that of film-based FETs, which is 0.61% (see Fig. S4 in Supplementary Information). When the temperature was varied from 280 K to 295 K, the Δ*R*/*R*_off_ gradually increased under each given gate bias, but suddenly became almost zero at 300 K in the metallic state. Figure 3b shows |Δ*R*/*R*_off_| as a function of gate bias for the nanowire and film-based devices near the phase-transition temperature. Δ*R*/*R*_off_ in the nanowire channel indicated greater sensitivity to increasing *V*_G_ than that of the film-based FETs. We also plotted the resistance modulation efficiency for VO_2_-based FETs with the various gate dielectrics reported to date, as shown in Fig. 3c. On the horizontal axis, the sheet carrier number induced by the gate dielectric Δ*n*_sheet_ (Δ*n*_sheet_ = *C*_i_ *V*_G_/*e*), where Electric field strength distribution._i_ is the capacitance per unit area and *e* is the elemental charge, was selected as the conventional model because of performance comparison of various VO_2_-based FETs. Therefore, devices that have low Δ*R*/*R*_off_ despite having high Δ*n*_sheet_ imply the existence of multiple charge trap levels. Among the devices of this type, the VO_2_ nanowire-based FET with the hybrid gate dielectric exhibits the high resistance modulation, despite a low sheet carrier density of approximately 5 × 10^12^ cm^−2^, compared with other solid-state VO_2_-based FETs^19–22^. Efficient resistance modulation was thus realized in nanowire-based FETs with hybrid gate insulators, though T. Yajima *et al*.^31^ reported the achievement of quite high resistance modulation due to carrier accumulation in non-conventional VO_2_ FETs using a depletion layer gate between VO_2_ and Nb-doped TiO_2_, as seen in the inset of Fig. 3c.

### Electric field strength analysis for the thin-film-based and nanowire-based devices

**Figure 4:**
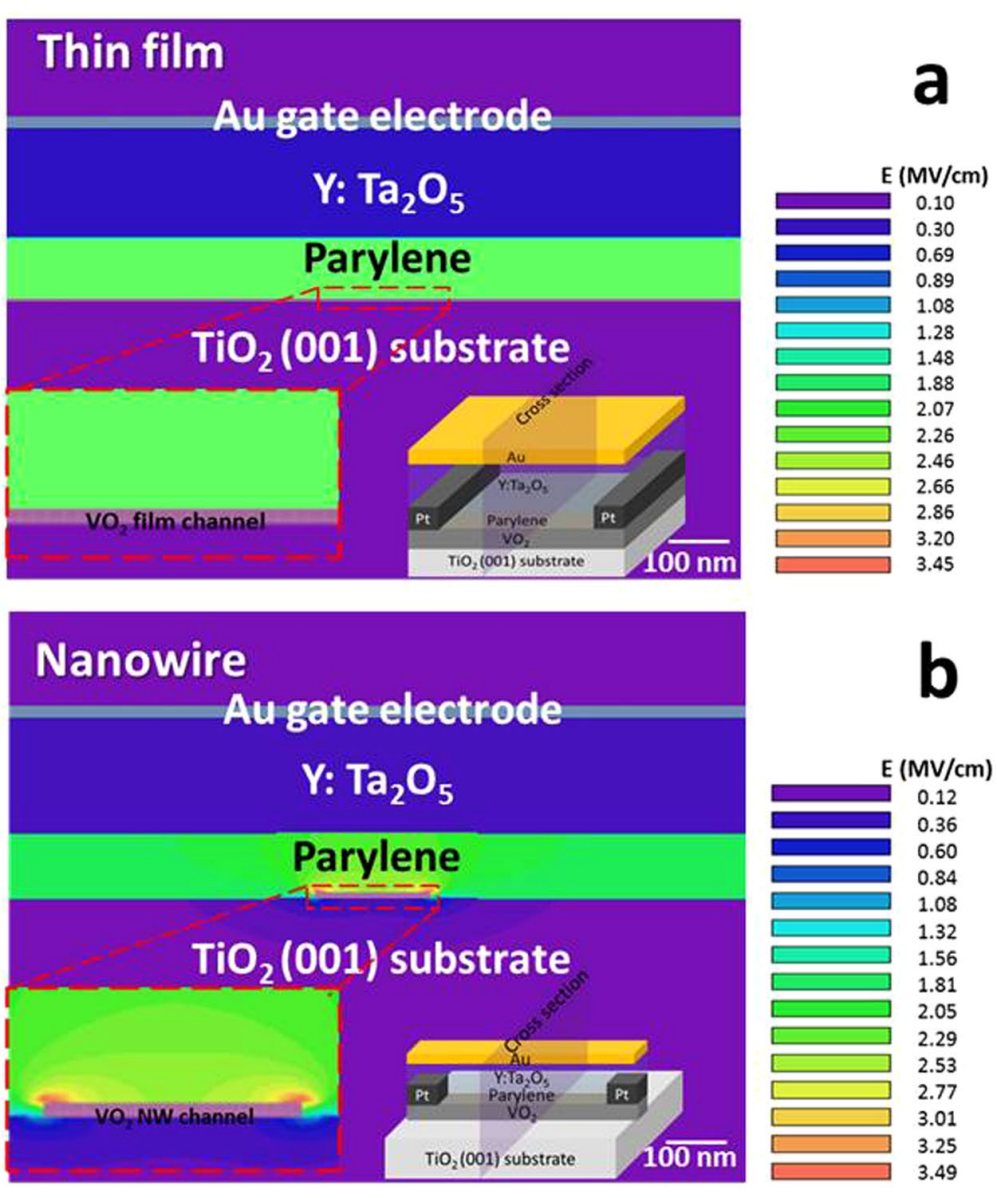
Electric field strength distribution. (**a**) and (**b**) Show the electric field distributions in the cross-sections of the VO_2_ nanowire and thin-film channels, respectively. The insets show the simulated device structures.

To clarify the origin of the enhancement in the resistance modulation in nanowire-based FETs, we first analysed the electric field strength distributions for the thin film-based and nanowire-based FETs using the finite element method (FEM), and the results are shown in Fig. 4a and b, indicating distinct differences. The electric field was uniformly distributed on the thin-film channel surface, as shown in the magnified image in Fig. 4a. In contrast, the nanowire-channel field distribution showed an arc shape (see the magnified image in Fig. 4b), and the prevailing field converged on the edges. The electric field strength at the edge of the nanowire channel was enhanced 1.5-fold over that of the thin-film channel. However, this difference in the magnitude of the electric field cannot fully explain the experimental result of the 10-fold higher resistance modulation ratio for nanowire-based FETs. This therefore indicated that the dominant factor for enhanced resistance modulation is not a variation in the number of accumulated carriers by applying a gate bias.

### Mechanism of the resistance modulation by applying *V*_G_

**Figure 5:**
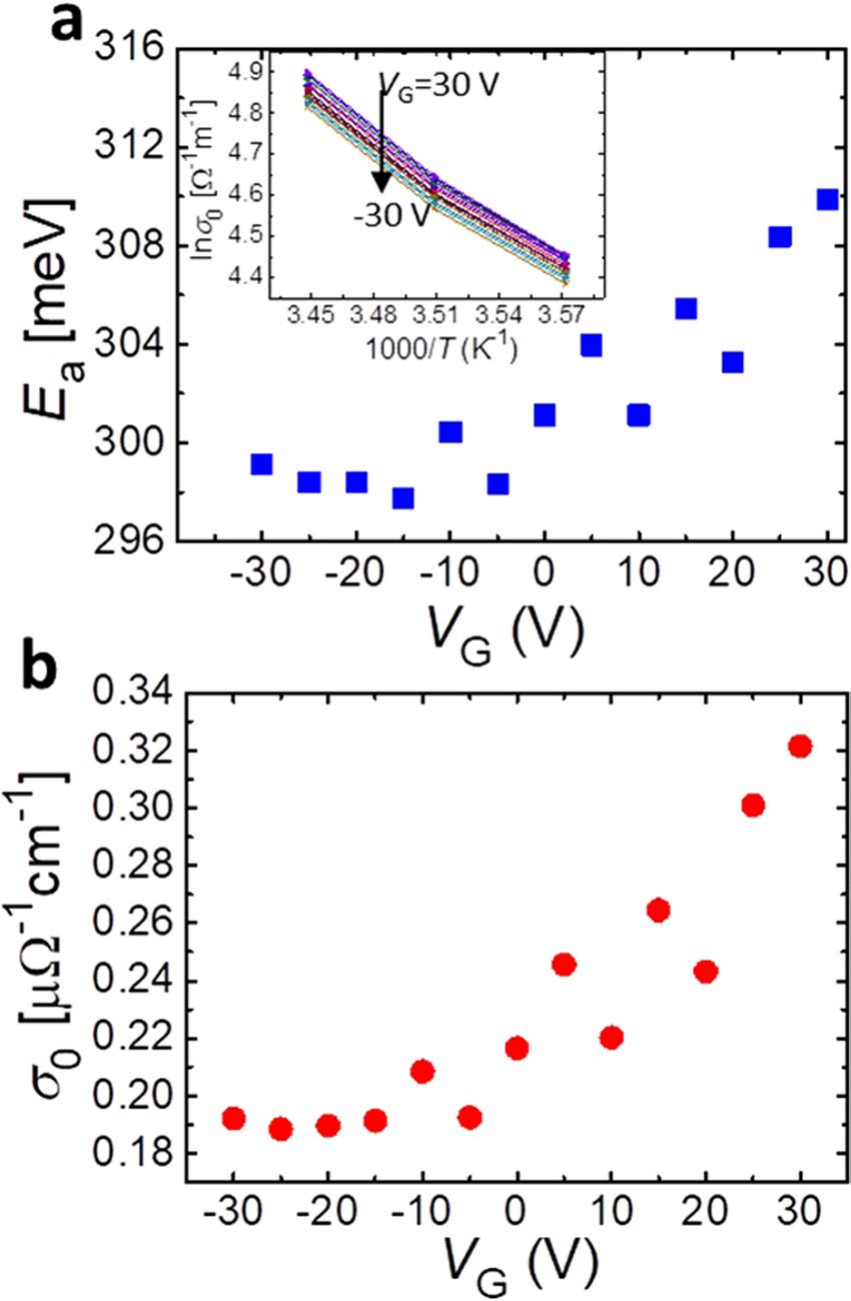
Mechanism of the resistance modulation by applying *V*_G_. Plot of (**a**), *E*_a_ and (**b**), σ_0_ versus gate bias for the nanowire-based FETs with Y-doped Ta_2_O_5_/parylene hybrid gate dielectrics, where *L* = 2 µm and *W* = 100 nm in the nanowire channel. The inset of Fig. 5a shows 1/*T* dependence of lnσ(*T*) with variety of *V*_G_ from −30 V to 30 V. Fittings by the least-square method were conducted to find the values of *E*_a_ and *σ*_0_. All of the correlation coefficients ${|{\rm{r}}|}^{2}$ of the fittings were over 0.99.

To provide a reasonable explanation for the resistance modulation in VO_2_ nanowire channels, we evaluated the activation energy for carrier hopping (*E*_a_) in an insulating state and the constant of transport conductivity (*σ*_0_) given by the following equation^32,33^.

2 $$\sigma (T)={\sigma }_{0}\exp (-\frac{{E}_{{\rm{a}}}}{{k}_{{\rm{B}}}T}),$$

where *k*_*B*_ is the Boltzmann constant. The natural logarithm conductance (ln*σ*(*T*)) from *V*_G_ = −30 V to 30 V as a function of 1/*T* are shown in the inset of Fig. 5a. The *E*_a_ and *σ*_0_ were derived by their linear fittings. Figure 5a and b show the *E*_a_ and *σ*_0_ behavior as a function of *V*_G_. In the positive *V*_G_, the both of *E*_*a*_ and *σ*_0_ increase with increasing *V*_G_. The change of *σ*_0_ means a change of coexistence state of inhomogeneity of metallic and thermally activated conductance in Si-based MOS-FET^34^. When this situation is applied to the VO_2_-based FET in this experiment, metallic nano-domains expand with increasing accumulated electron carriers due to applying positive *V*_G_, especially at the edge part of VO_2_ nanowire channel that an electric field converges as seen in Fig. 4b in a coexistent state of metallic and insulating phases near the *T*_MI_. Regarding the *E*_a_ behavior in the positive *V*_G_ region, the value increases approximately 12 meV from 298 meV to 310 meV, which is a factor of decrease in transport conductivity. However, influence of the increase in *E*_a_ is small near room temperature because thermal activation energy at room temperature is approximately 30 meV, which is over 12 meV. Thus the main factor of decrease in resistance by positive *V*_G_ is due to expansion of metallic nano-fractions by increasing *σ*_0_ in an insulating matrix. In the region of negative *V*_G_, the values of *E*_a_ and *σ*_0_ are almost constant, meaning that the negative *V*_G_ doesn’t affect the activation energy and the coexistence state regardless of the increase in resistance as shown in Fig. 2 and Fig. 3a. Thus, the resistance enhancement by negative *V*_G_ would be due to formation of depletion layer at the interface between VO_2_ channels and gate insulating layers.

## Discussion

In conclusion, superior resistance modulation sensitivity was obtained in VO_2_ nanowire-based FET devices by reducing the channel to the nanoscale (100 nm). Additionally, the maximum resistance modulation efficiency was observed near the transition temperature. We proposed that the enhanced resistance modulation of the nanowire channels was primarily due to the expansion of metallic nano-fractions in an insulating matrix especially at the edge parts of channel that an electric field converses. These results provide insight into the underlying properties of coexistence states of strongly correlated oxides and suggest possibilities for using nanoscale devices in high-performance next-generation electronics.

## Methods

### Nanowire synthesis

VO_2_ nanowires were synthesized by nanoimprint photolithography (NIL), followed by the fabrication of epitaxial VO_2_ thin films, which were grown on TiO_2_ (001) single-crystal substrates by pulsed laser deposition using an ArF excimer laser at 450 °C under an oxygen pressure of 1.0 Pa. Oxygen reactive ion etching (RIE) was then performed at a power of 50 W, a pressure of 4 Pa, and a flow of 70 sccm for 150 s to remove the resist residue from the compressed regions. Subsequently, SF_6_ gas was used for VO_2_ etching (60 W, 1.0 Pa, 10 sccm, 10 s).

### Device fabrication

VO_2_ thin-film-based FETs were fabricated by the stencil mask method, as reported in the literature. Because the stencil mask approach is restricted to hundreds of microns, conventional photolithography was substituted for the stencil mask to form the standard three-terminal electrode pattern for nanowire-based FETs. Pt (20 nm)/Cr (5 nm) source and drain contacts were sputtered with 2 µm spacing. Subsequently, a hybrid gate insulator consisting of the high-*k* inorganic oxide Ta_2_O_5_ and parylene organic polymer was fabricated, as reported in detail elsewhere^27^. A 50-nm-thick Au film was deposited as the gate electrode by electron-beam evaporation. The channel area overlap between the nanowires and the Au gate electrode was 100 nm × 2 µm.

### Measurements

All electrical measurements were performed using an apparatus with a source measure unit (2635A, 236, Keithley), in which the attached Peltier-based sample stage was responsible for temperature control. Nitrogen gas was introduced to protect the VO_2_ surface from humidity^35^ and water^36^.

## Electronic supplementary material

Supplementary Information
