# Supplementary material for: Enhanced electronic-transport modulation in single-crystalline VO2 nanowire-based solid-state field-effect transistors
Source: Sci Rep. 2017 Dec 8;7:17215. doi: 10.1038/s41598-017-17468-x (PMC5722937; doi:10.1038/s41598-017-17468-x)
Supplement: Supplementary file 1 — Supplementary Information [file 41598_2017_17468_MOESM1_ESM.pdf]

SUPPLEMENTARY INFORMATION

Enhancement of electronic transport modulation in single crystalline VO_2 nanowire-based solid-state field-effect transistor

Tingting Wei [1,2], Teruo Kanki [1,*], Masashi Chikanari [1], Takafumi Uemura [1], Tsuyoshi Sekitani [1] and Hidekazu Tanaka[1,*]

[1] *Institute of Scientific and Industrial Research, Osaka Universit, Ibaraki, Osaka 567-0047, Japan*

[2] *Faculty of Science, Kunming University of Science and Technology, Kunming 650093, China*

**E-mail: kanki@sanken.osaka-u.ac.jp, h-tanaka@sanken.osaka-u.ac.jp*

Supplementary figures and captions

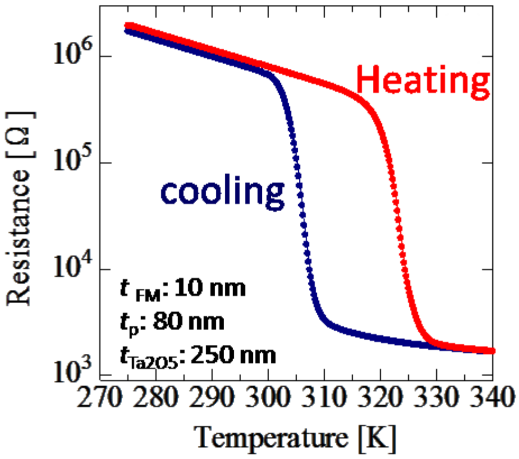

Figure S1. (Color online) Resistance as a function of temperature during heating (red line) and cooling (blue line) for thin film-based FETs with Y:Ta_2O_5/parylene hybrid gate dielectrics.

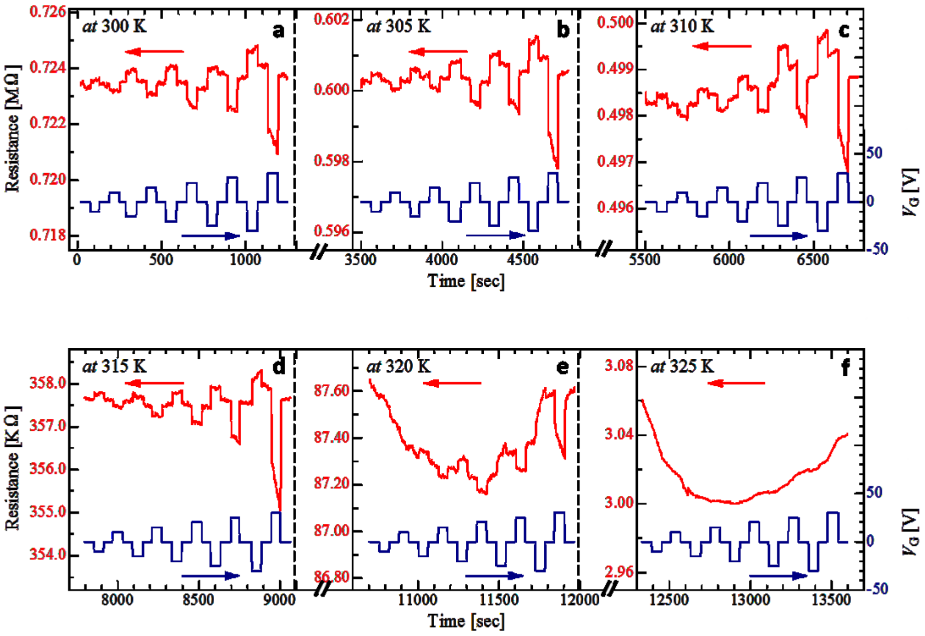

Figure S2. (Color online) (a–f) Resistance response to gate bias in VO_2 thin film-based FET with hybrid gate insulator near T_{MI} regime from 300 K to 325K.

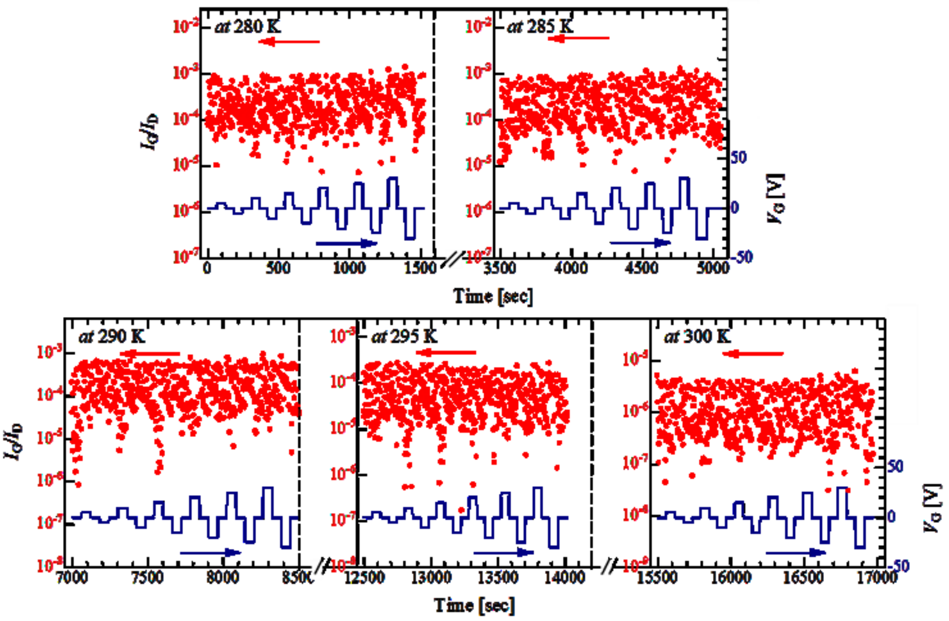

Figure S3. (Color online) Ratio of leakage current I_G to source-drain current I_D plotted against t measurement time for VO_2 nanowire-based FET with hybrid gate insulator. Ratios as low as 10^{-3} indicated that the device remained unbroken during the measurement process.

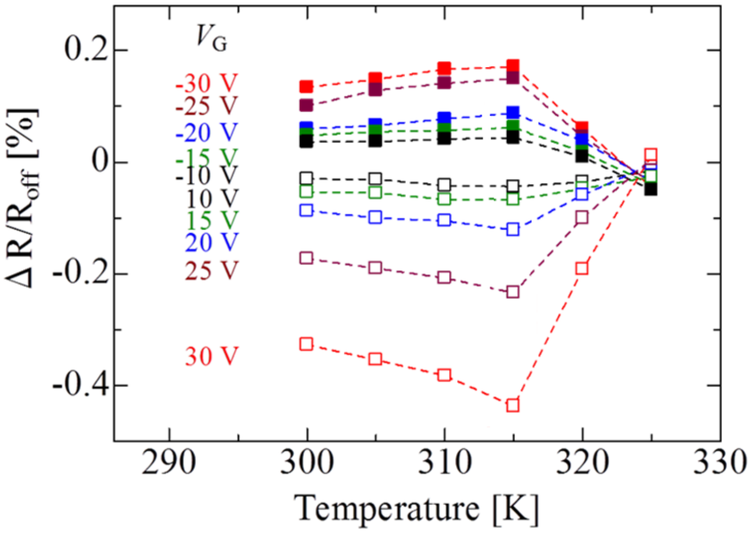

Figure S4. (Color online) Temperature dependence of resistance modulation efficiency at various gate biases for hybrid gate insulator-gated VO_2 thin film FET.

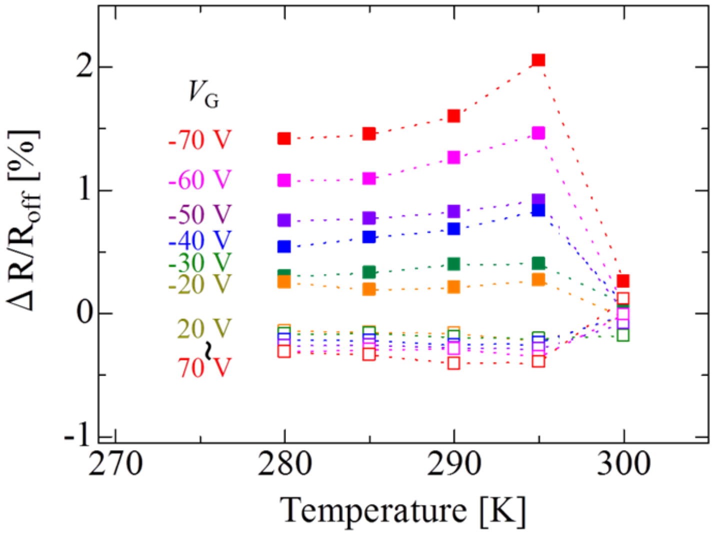

Figure S5. (Color online) Resistance modulation ratios versus temperature for VO_2 nanowire-based FET with 300-nm-thick parylene monolayer gate insulator. The maximum value was 2.44% at ±70 V. When the result was related to the induced sheet carrier density, the difference between the parylene-C monolayer gate insulator and the hybrid gate insulator (four times) can be explained based on the different dielectric constants, similar to the case in thin film-based devices[S1].

[S1] T. Wei, T. Kanki, K. Fujiwara, M. Chikanari, H. Tanaka, *Appl. Phys. Lett.* **108**, 053503 (2016).
